# No Association between staging operation and the 5-Year Risk of Reoperation in Patients with Crohn’s Disease

**DOI:** 10.1038/s41598-018-34867-w

**Published:** 2019-01-22

**Authors:** Jiajie Zhou, Yi Li, Jianfeng Gong, Weiming Zhu

**Affiliations:** 1 Department of General Surgery, Jinling Clinical Medical College, Nanjing Medical University, Nanjing, Jiangsu 210002 China; 2 Department of General Surgery, Huai’an First People’s Hospital, Nanjing Medical University, Huai’an, Jiangsu 223300 China

## Abstract

The aim of this study was to investigate the impact of staging operation on the risk of reoperation in patients with CD who underwent primary bowel resection. This was a retrospective study of 980 patients with CD who were hospitalized in Jinling Hospital Affiliated to Nanjing Medical University between January 1, 2001, and October 1, 2016. The patients were grouped according to staging operation (n = 64) and one-stage operation (n = 148). Postoperative intestinal function recovery time, postoperative short-term complications, and reoperation rates were compared between the two groups. There was significant difference in disease behavior between the staging operation group and the one-stage operation group. There was no significant difference in postoperative tolerance of enteral nutrition among groups (P > 0.05). Obvious differences were found in the comparison of the first time of exhaustion, defecation after operation, postoperative length of stay and postoperative complications among groups (all P < 0.05). There was no difference in the 5-year cumulative reoperation-free rates between the two groups (P > 0.05). In conclusion, surgical intervention at proper time and appropriate operation during operation are essential for patients with CD. It is believed that staging operation with ostomy followed by intestinal anastomosis is feasible when there are more than two risk factors for postoperative intra-abdominal infectious complications.

## Introduction

Crohn’s disease (CD) is a chronic inflammatory granulomatous disease of the intestines with lifelong relapse. Even though internal medicine is the major therapeutic approach, 70~90% of the disease requires surgical intervention eventually^1–3^. Surgical intervention is used frequently for intestinal stenosis, perforation, abdominal abscess and other complications caused by recurrent intestinal inflammation. CD patients required surgical treatment not only have CD complications that need to be managed by surgery, but also suffer from malnutrition caused by long-term intestinal dysfunction and immune disorders due to long-term administration of drugs. Some patients even require emergency surgery that significantly limit adequate perioperative preparation.

The principle of surgical treatment for CD is to control the symptoms, and corresponding operative modes include diseased intestinal resection, ostomy and drainage of abdominal abscess, etc. When the whole-body situation of the patient is not allowed to perform major incision, surgical treatment in accordance with the concept of “damage control surgery (DCS)” can reduce the risk of treatment to a certain extent and improve corresponding clinical effect. In 1990s, Rotondo^4^ formally put forward the concept of DCS, and expounded the three-stage principle of DCS, including fast control of the damage, resuscitation, and definitive operation. For critically ill patients with CD, under the guidance of the DCS concept, staging surgery is commonly adopted, with the performance of one-stage ostomy to deal with intestinal complications possibly, and restore intestinal function rapidly, so as to improve the nutritional status of the patients. Furthermore, definitive operation will be carried out on the basis of general condition improvement in the patients. Staging operation is a common approach for CD patients. Nevertheless, there is no relevant literature so far to verify whether the risk of reoperation in these patients is increased with staging operation compared with one-stage definitive operation.

Therefore, the present study was conducted to explore the effect of staging operation on postoperative reoperation rate of CD patients. The results of this study may be beneficial for optimizing the therapeutic regimen and improve the quality of life in CD patients postoperatively.

## Materials and Methods

### Study Design and Patients

This study was approved by the ethics committee of Jinling Hospital Affiliated to Nanjing Medical University. The need for individual consent was waived by the committee because of the retrospective nature of the study. This was a retrospective study of 980 patients with CD who were hospitalized in Jinling Hospital Affiliated to Nanjing Medical University between January 1, 2001, and October 1, 2016.

Inclusion criteria of eligible patients: (1) The diagnosis and treatment of CD were carried out based on the European Crohn’s and Colitis Organization (ECCO) consensus standard^5,6^; (2) CD patients with one-stage resection and intestinal anastomosis for the first time in our hospital; (3) Patients with staging operation who underwent ostomy in our hospital for the first time, as well as two-stage ostomy and intestinal anastomosis. The exclusion criteria were: (1) incomplete medical records; or (2) Patients who had enteric resection and enterostomy in other hospital prior to admission.The diagnosis and treatment of all patients were accordant with the recommended standard of ECCO consensus.

**Figure 1:**
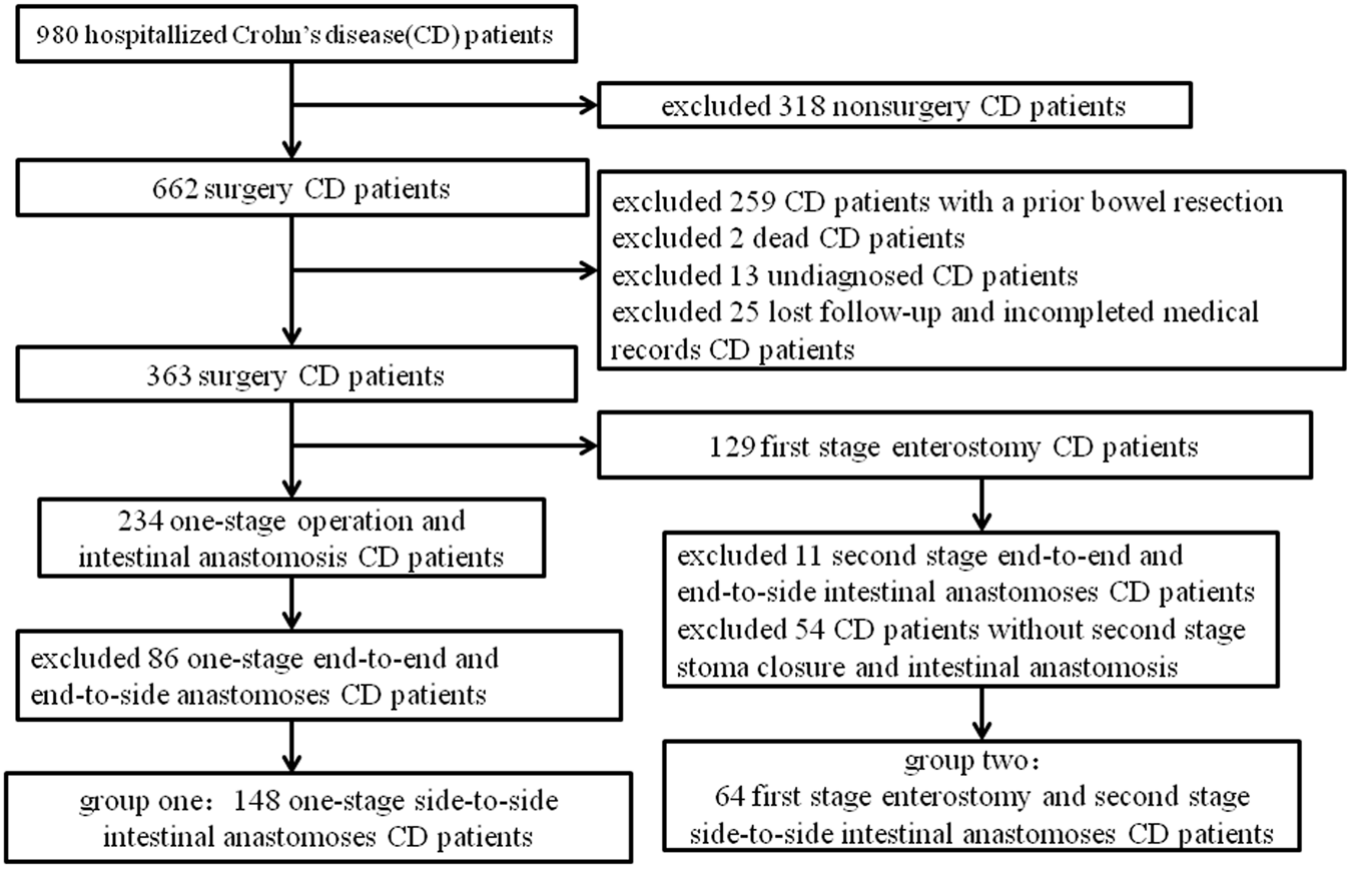
Study selection flow chart.

The strategy used on selected patients was shown in Fig. 1. A total of 148 CD patients underwent one-stage stapled side-to-side anastomosis (group one) and 64 CD patients with first stage enterostomy and second stage side-to-side anastomoses (group two) were included in the final analysis.

**Figure 2:**
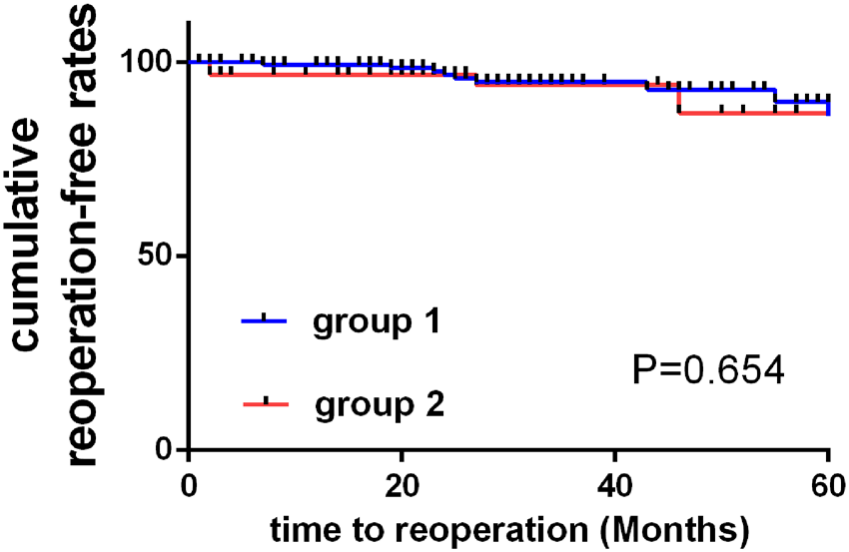
Kaplan-Meier curves of cumulative reoperation-free rates in the two groups are shown in Fig. 2.

Group one patients underwent primary intestinal resection with one-stage stapled side-to-side anastomosis. cover stoma was not allowed on this group. All Group two patients received two-stage operation. A temporary stoma might be adopted for these patients with emergency visit, poor nutritional status or risk factors for postoperative abdominal infection at first stage. Besides, during the first stage operation, intestinal lesions would be treated simultaneously or with the performance of exclusion surgery at the same time, and cover the stoma at second stage operation with or without definitive operation. CD patients with permanent stoma after the first stage operation were excluded from the study. Only disease recurrence related operations were considered reoperations.

Indications for two-stage operations included acute diffuse peritonitis and two or more risk factors. Risk factors for postoperative abdominal infection included poor preoperative nutritional status (preoperative albumin levels <35 g/l), preoperative hormone use, preoperative intraperitoneal abscess, preoperative immunosuppressive agents and biological agents.

Group one patients and Group two patients before second stage operation needed to improve nutritional status (preoperative albumin levels >35 g/L) and induce disease remission (c-reactive protein <8 mg/L) preoperation by enteral nutrition, abdominal abscess drainage and anti-infection treatment.

### Pre- and Perioperative Management

All patients underwent perioperative management according to the enhanced recovery after surgery model (ERAS)^7,8^, including no mechanical bowel preparation, drinking water 2 h before surgery, having solid diet 6 h before surgery, preoperative non-prophylactic use of intestinal antibiotics, intraoperative limiting of fluid intake (less than 1500 ml), postoperative non-routine gastric tube implantation, early pulling out urinary catheter, early oral diet and out of bed activity, prohibition of opioid analgesics, and postoperative limited fluid therapy (according to blood lactate level). In group one, 63 patients were treated with azathioprine preoperation. 6 patients received anti-TNF drugs before operation. In group two, 41 patients were treated with azathioprine before second stage operation. 3 patients received anti-TNF drugs before second stage operation.

### Postoperative Management

All patients were treated with enteral nutrition early in the postoperative period, and administrated with azathioprine (1.5 mg/kg/d) orally within two weeks after operation. Medication would be replaced if the patients could not tolerate azathioprine. Detailed information related to postoperative medication was described in Table 1.

**Table 1:** Characteristics of the Patients.

Variables	Group 1 (n = 148)	Group 2 (n = 64) first stage	P
Female	91 (61.5%)	49 (76.6%)	0.591
Duration of disease (months)	35 (1–226)	33 (1–243)	0.579
**Prior surgery history**			
Abdominal abscess drainage	35 (23.6%)	10 (61.5%)	0.190
Appendectomy	28 (18.9%)	12 (15.6%)	0.977
Perianal operation	26 (17.6%)	14 (21.9%)	0.462
Smoking history	9 (6.1%)	5 (7.8%)	0.641
**Surgical approach**			
Laparoscopic/open	58/90	8/56	<0.001
**Timing of operation**			
emergency/selective	1/147	9/55	<0.001
**Montreal Classification at diagnosis**			
Age of diagnosis			0.713
A1 (≤16 years)	11 (7.4%)	8 (12.5%)	
A2 (17–40 years)	107 (72.3%)	42 (65.6%)	
A3 (>40 years)	30 (20.3%)	14 (21.9%)	
Location of disease			0 0.363
L1 (Terminal ileum)	69 (46.6%)	26 (40.6%)	
L2 (Colonic)	9 (6.1%)	13 (20.3%)	
L3 (Ileocolonic)	44 (29.7%)	24 (37.5%)	
L4 (Upper GI)	26 (17.6%)	1 (1.6%)	
Behavior of disease			<0.001
B1 (Non-str/non-pen)	8 (5.4%)	5 (7.8%)	
B2 (Stricturing)	85 (57.4%)	16 (25%)	
B3 (Penetrating)	55 (33.5%)	43 (67.1%)	
P (Perianal lesions)	34 (29.8%)	20 (31.2%)	0.204
Postoperative management			0.253
Mesalazine	13 (8.8)	2 (3.1%)	
Azathioprine	115 (77.7%)	48 (75%)	
Remicade	2 (1.4%)	2 (3.1%)	
Tripterygium glycosides	17 (11.5%)	10 (15.6%)	
Thalidomide	1 (0.7%)	2 (3.1%)	

GI: gastointestinal; non-str: non-stricturing; non-pen: non-penetrating.

Group A: one-stage operation group; Group B: staging operation group.

### Data Collection

The clinical data were collected from the hospital database, including the patient’s general situation, preoperative and postoperative medication, surgical records, Montreal classification, and reoperation. The outpatient and inpatient records of all CD patients were retrospectively analyzed. As per out institution’s standard, all patients with CD are followed up at the outpatient clinic after surgery. If follow-up made at another hospital or if a visit is skipped, telephone follow-up is done. Patients who were nonetheless lost to follow-up were censored at their last visit.

Montreal typing^9^ was used to describe the patient’s age at diagnosis, location of the disease, and disease behavior. According to the age of diagnosis, patients were divided into three grades: (A1) < 16 years old; (A2) 17–40 years old; and (A3) > 40 years old. Patients were classified into: (L1) ileum; (L2) colon; (L3) colon; and (L4) upper alimentary tract based on the location of the disease. In accordance with disease behavior, patients were divided into: (B1) non-stenotic and non-penetrating lesions; (B2) stenotic lesions; (B3) penetrating lesions; and (P) perianal lesions.

The staging operation referred to an operation plan that a temporary ostomy might be firstly adopted for patients with emergency visit, poor nutritional status or risk factors for postoperative abdominal infection. Besides, during the operation, intestinal lesion would be treated simultaneously or with the performance of exclusion surgery at the same time, and definitive operation would be performed during two-stage ostomy and intestinal anastomosis. In addition, the one-stage operation was defined as the one-stage resection of the intestinal canal and the side-to-side anastomosis of the intestine following adequate perioperative preparation, improvement of nutritional status in patients, and normal preoperative maintenance of inflammation indexes.

### Outcomes

To group one (one-stage operation) CD patients, follow-up started on the initial operation date.To group two (staging operation) CD patients, follow-up started on the second stage operation date. The primary study outcome was reoperation during follow-up. The disease duration was defined as the time from diagnosis to initial surgery. Perioperative complications were observed according to the Dindo-Clavien classification criteria^10^: grade I: without requirement of other treatment in addition to the antiemetic, antipyretic, and analgesic agents, diuretic, and electrolyte, physiotherapy, or bedside opening of infected wounds; grade II: need for medical treatment, antibiotic treatment for incision infection, or need for blood transfusions and total parenteral nutrition; grade III: need for surgical, endoscopic, or interventional radiology treatment; grade IV: requirement of intermittent monitoring or transfer to the ICU for life-threatening complications (including central nervous system complications); and grade V: death.

### Statistical Analysis

All data were analyzed using SPSS 21.0 (IBM, Armonk, NY, USA). Continuous data distribution was determined using the Kolmogorov-Smirnov test. Normally distributed data were presented as mean ± standard deviation and analyzed using the Student t test. Non-normally distributed data were presented as median (range) and analyzed using the Mann-Whitney U test. Categorical data were presented as frequencies and analyzed using the chi-square or Fisher exact test, as appropriate. The cumulative reoperation rate was calculated using the Kaplan-Meier method and analyzed using the log-rank test. Two-sided P-values < 0.05 were considered statistically significant.

## Results

### Characteristics of the Patients

As shown in Table 1, there was no difference in terms of gender, course of disease, preoperative history of operation, age of diagnosis, location of disease and medication after operation between groups. Furthermore, obvious differences were observe regarding operation mode, operation timing and disease behavior between groups. Meanwhile, rates for laparoscopic operation and elective surgery were higher in the one-stage operation group than those in the staging operation group, while the proportion of penetrating lesions was lower than that in the latter group. However, the proportion of stenotic lesions was higher in the one-stage operation group than that of the staging operation group. The median time of ostomy and intestinal anastomosis was 7 months (3–24) in the staging operation group.

### Comparison of Postoperative Observation Indexes between Groups

As illustrated in Table 2, there was no significant difference in postoperative tolerance of enteral nutrition among groups (P > 0.05). Obvious differences were found in the comparison of the first time of exhaustion, defecation after operation, postoperative length of stay and postoperative complications among groups (all P < 0.05). Compared with patients in the one-stage operation group and patients with ostomy and intestinal anastomosis in the staging operation group, the postoperative exhaustion and defecation time were much earlier in patients with ostomy of the staging operation group. Nevertheless, the time of postoperative length of stay was longer in patients with ostomy of the staging operation group than those of the former two groups. Furthermore, in patients with ostomy of the staging operation group, postoperative grades II, III and IV complications were higher than those of the other two groups. Moreover, there was no IV complication in the latter two groups. After ostomy in the staging operation group, there were 5 cases of IV complication, 3 cases of septic shock, 1 case of anastomotic bleeding and 1 case of cardiac insufficiency, all of which were improved after supportive treatment when transferring to the ICU.

**Table 2:** Postoperative observation indexes.

Postoperative observation indexes	Group1 (n=148)	Group 2 (n=64)	Group 2 (n=64)	P
		first stage	second stage	
Time of exhaustion (days)	3 (1–8)	2 (1–13)	3 (2–7)	<0.001
Time of defecation (days)	3 (1–9)	2 (1–13)	3 (2–7)	<0.001
Tolerance period of enteral nutrition (days)	4 (2–20)	4 (1–15)	4 (2–10)	0.653
Postoperative hospital stay (days)	6 (3–33)	9 (3–56)	7.5 (3–25)	0.021
**Postoperative complications**				0.013
**Grade I**	20 (13.5%)	6 (9.3%)	12 (18.8%)	
Abdominal distension	4 (2.7%)	2 (3.1%)	2 (3.1%)	
Infection of incisional wound	8 (5.4%)	4 (6.3%)	9 (14.1%)	
Diarrhea	7 (4.7%)			
Hydrothorax	1 (0.7%)		1 (1.7%)	
**Grade II**	28 (18.9%)	24 (37.5%)	18 (28.1%)	
Intestinal obstruction	1 (0.7%)	1 (1.6%)		
Incision bleeding	1 (0.7%)	1 (1.6%)		
Fungal infection		1 (1.6%)		
Abdominal distension	12 (8.1%)	4 (6.3%)	11 (17.2%)	
High-output stoma		7 (10.9%)		
Anemia	6 (4.1%)	10 (15.6%)	1 (1.6%)	
Anastomotic bleeding	1 (0.7%)		1 (1.6%)	
Diarrhea	5 (3.4%)		5 (7.8%)	
Intra-abdominal infection	1 (0.7%)			
Pulmonary infection	1 (0.7%)			
**Grade III**	9 (6.1%)	7 (10.9%)	4 (6.3%)	
Duodenal fistula		1 (1.6%)		
Cholecystitis		1 (1.6%)		
Pleural effusion		3 (4.7%)	1 (1.6%)	
Hydrops abdominis	2 (1.4%)	2 (3.1%)		
Anastomotic fistula	4 (2.7%)		1 (1.6%)	
Intra-abdominal hemorrhage	1 (0.7%)			
Incision hemorrhage	1 (0.7%)		1 (1.6%)	
Intra-abdominal abscess	1 (0.7%)		1 (1.6%)	
**Grade IV**		5 (7.8%)		
Infectious shock		3 (4.7%)		
Anastomotic hemorrhage		1 (1.6%)		
Cardiac failure		1 (1.6%)		

### Comparison of Cumulative Reoperation-Free Rates

No patient died during the follow-up period. In group one (148 patients), 11 patients (7.43%) needed reoperation due to disease recurrence after primary operation during the follow-up period. In group two (64 patients), 4 patients (6.25%) as well as required reoperation because of disease recurrence after second stage operation during the follow-up period. The 5-year cumulative reoperation-free rate of the two groups was illustrated in Fig. 1. There was no statistically significant difference in reoperation-free rate between groups within 5 years (P = 0.654).

## Discussion

In this study, there was no difference in gender, course of disease, previous surgical history, age of diagnosis, location of the disease, and postoperative medication in the observed patients between groups. Moreover, significant differences were observed between groups regarding operation mode, operation timing and disease behavior. The above results might be attributed to the selection of treatment plan for CD patients in this IBD center. Intraperitoneal lesions was evaluated by hematopoietic, endoscopic and imaging examinations. For patients with one-stage operation of definitive operation, adequate perioperative management was carried out, including perioperative nutritional support and nutritional status improvement. Abdominal puncture and drainage were performed in patients with abdominal abscess. After preoperative reduction of inflammatory indexes levels in the blood to the normal range, the lesion was resected with one-stage anastomosis. Furthermore, for patients undergoing emergency operation and with poor nutritional status or severe intraoperative abdominal contamination, staging operation was adopted by using one-stage temporary ostomy and two-stage intestinal anastomosis. In addition, for patients who required temporary ostomy, abdominal abscess drainage or diseased intestinal resection would be performed during operation, and enteral nutrition support was given early after operation. Prior studies^11^ documented that perioperative nutritional support could not only reduce the incidence of postoperative complications, but also induce CD remission, eliminate intestinal obstruction, promote spontaneous closure of enterocutaneous fistula and avoid another risk of surgery. As proved in the study conducted by Gutierrez^12^, in CD patients required surgery, the abscess drainage should be performed firstly when combining with the formation of abscess, and some patients no longer needed surgical treatment after abscess dissipation. The first choice for abscess drainage was percutaneous puncture and drainage^13^. In such way, some perforated lesions of the intestines could be healed and the possibility of intestinal resection could be reduced accordingly^14^. Besides, the risk of operation could be significantly decreased simultaneously even if it was necessary to remove the penetrating ulcer.

Previous evidence^15^ suggested that intestinal fistula, abdominal infection, malnutrition and other risk factors were potential risk factors for postoperative complication of abdominal infection. Intra-abdominal septic complications (IASC) (Anastomotic leakage, abdominal abscess, or peritonitis) were risk factors for postoperative recurrence of the disease^16^. A Study^17^ has shown that nearly 50% of patients required reoperation due to postoperative complications in patients with abdominal cavity infection. It was indicated that during staging operation, the application of temporary ostomy could improve the safety of operation and reduce the incidence of IASC remarkably^15,17–19^. At present, postoperative IASC risk factors are considered to include poor preoperative nutritional status, preoperative hormone use, preoperative intraperitoneal abscess, preoperative immunosuppressive agents and biological agents, etc^15,16,19^. Furthermore, as verified by past studies, with the increase of risk factors, the incidence of postoperative IASC would be increased at the same time. Moreover, the incidence of postoperative complications would elevate to 40% when there was preoperative hormone use history and abdominal abscess^19^. Therefore, during preoperative evaluation, it is recommended that staging operation with ostomy and intestinal anastomosis should be performed primarily if there are two or more risk factors in the target patients. The study^17^ has also discovered that the first implementation of ostomy could significantly reduce risk factors of the operation and incidence of postoperative complications, without clear impact on the overall number of operations and postoperative length of stay, which was consistent with the results of our study. In addition, over 75% of patients could recover intestinal continuity^20^, besides, the median time of ostomy and intestinal anastomosis was about 5 months. In the present study, the median time of ostomy and intestinal anastomosis was estimated to be 7 months.

Our study found that Group two patients at the first stage operation had higher proportion of penetrating diseases and emergency surgery than one-stage operation patients. The literature^5,6^ shows that penetrating lesions and emergency surgery are risk factors for reoperation, but our study found no difference in long-term reoperation rates between one-stage and two-stage operation groups. We thought two-stage operation patients had poor nutritional status and disease active at their first stage operation. This situation led to their proportion and grade of postoperative complications were significantly higher than one-stage operation patients. Due to the first stage operation, their nutrition and infection status had been better at the second stage of opertion. When staging operation patients received the second stage operation, they were under the similar condition to surgery, resulting in no difference in long-term reoperation rates between two groups.

In the present study, the postoperative length of stay, as well as the proportion and level of postoperative complications were estimated to be higher for patients with ostomy in the staging operation group than those in the one-stage operation group. Possible reason might be attributed to the poor general condition and critically ill situation of patients who required ostomy. However, there was no difference in postoperative exhaustion and defecation, early postoperative enteral nutrition support, postoperative length of stay, and postoperative complications in patients undergoing ostomy and intestinal anastomosis in the staging operation group when compared to those in the one-stage operation group.

The advantages of ostomy were early postoperative exhaustion and defecation, rapid providing of enteral nutrition, and improvement in the nutritional status of patients, and there was no risk of anastomotic leakage. Existing literature^21–23^ showed that fecal passage diversion could significantly improve the inflammation of distal indwelling colon. The “sutures with mucosal eversion” technique^24^ greatly reduced the incidence of complications of ostomy in our study. Besides, no complications such as stomal stenosis, para-stomal hernia and stoma necrosis occurred after ostomy and during intestinal anastomosis. However, about 11% of patients underwent ostomy appeared water-electrolyte disturbance due to postoperative excessive loss of ostomy fluid, which were eventually improved after timely supportive treatment. Studies^25^ have evaluated that the incidence of water-electrolyte disturbance was the highest in CD patients after ostomy, accounting for 29% of all complications. Besides, loop ileostomy was the main risk factor for patients with terminal ileostomy. In those patients^26^, the intake and discharge of liquid should be monitored closely, in combination with the restriction of the intake of hypotonic solution. If necessary, isotonic liquid, antidiarrheal agents and acid inhibitors should be provided in those patients, so as to ensure the balance of intake and discharge.

The existing data supported that smoking history^27,28^, penetrating lesions^27,29^, postoperative perianal lesions^28,29^ and emergency surgery^28^ were risk factors for postoperative recurrence, whereas azathioprine^27^, antitumor necrosis factor^29^ and mesenteric resection^30^ were protective factors. Due to the long-term use of glucocorticoids, immunosuppressive agents and malnutrition in patients required surgical treatment, one-stage temporary ostomy to improve nutritional status, as well as two-stage ostomy and intestinal anastomosis were common modes of operation under these circumstances. However, there is no literature review about whether the recurrence rate of CD patients is increased under staging operation compared with that of the one-stage definitive operation. In our study, there was no difference in the long-term reoperation rate between the staging operation and the one-stage definitive operation, and the operative complication was relatively low during intestinal anastomosis. Therefore, staging surgical program is recommended for patients with poor nutritional status, emergency operation and risk factors of postoperative intraperitoneal infectious complications.

There are some limitations to this study. Firstly, this study is a retrospective analysis of medical records, with certain bias of the data. Secondly, our Inflammatory Bowel Disease (IBD) Center is the only specialized IBD clinic in China, and the patients who are being transferred to our hospital have serious symptoms and complicated conditions. Thirdly, patients who underwent primary bowel resection in another hospital were excluded, leading to a small sample size. Nevertheless, this study is the first to explore the effect of residual lesions on reoperation rates after surgery for CD. A prospective, large sample study is needed to validate the conclusions.

In conclusion, surgical intervention at proper time and appropriate operation during operation are essential for patients with CD. It is believed that staging operation with ostomy followed by intestinal anastomosis is feasible when there are more than two risk factors for postoperative intra-abdominal infectious complications. In addition, regular follow-up and medical treatment are of critical importance for CD patients.
